# A novel small-molecule compound S-342-3 effectively inhibits the biofilm formation of *Staphylococcus aureus*

**DOI:** 10.1128/spectrum.01596-23

**Published:** 2023-10-11

**Authors:** Jiao Zhang, Li Shen, Peiyao Zhou, Shuying Chen, Bingjie Wang, Cailin Wan, Weihua Han, Lulin Rao, Huilin Zhao, Xinyi Wang, Chunyang Wu, Junhong Shi, Yanghua Xiao, Zengqiang Song, Fangyou Yu, Chunchan Lin

**Affiliations:** 1 Department of Clinical Laboratory, Key Laboratory of Clinical Laboratory Diagnosis and Translational Research of Zhejiang Province, The First Affiliated Hospital of Wenzhou Medical University, Wenzhou, Zhejiang, China; 2 Department of Clinical Laboratory Medicine, Shanghai Pulmonary Hospital, Tongji University School of Medicine, Shanghai, China; 3 Jiangxi Provincial Key Laboratory of Preventive Medicine, School of Public Health, Nanchang University, Nanchang, China; 4 Department of Respiratory Medicine, The First Affiliated Hospital of Wenzhou Medical University, Wenzhou, China; 5 School of Pharmaceutical Sciences, Wenzhou Medical University, Wenzhou, China; Icahn School of Medicine at Mount Sinai, New York, New York, USA

**Keywords:** *Staphylococcus aureus*, biofilm, small-molecule, PIA, cell adhesion

## Abstract

*Staphylococcus aureus* is an important human pathogen in both community and hospital settings that often causes persistent and recurrent infections. The continuous emergence of multidrug-resistant strains and the lag in antibiotic development make the treatment of *S. aureus* more challenging. The biofilm formation of *S. aureus* is an important reason for persistent infection. In this study, we demonstrated that a small-molecule compound S-342-3 can effectively inhibit the biofilm formation of *S. aureus*. The crystal violet semi-quantitative assays revealed that at a sub-minimum inhibitory concentration of 4 µg/mL, S-342-3 reduced *S. aureus* biofilm mass by 57.43%, 52.14%, and 25.49%. Using confocal laser scanning microscopy, we observed that the biofilm was more incompact and less uniform upon the treatment of S-342-3. At a sub-inhibitory concentration (4 µg/mL), the S-342-3 can effectively reduce the production of polysaccharide intercellular adhesin (PIA) and suppress the initial adhesion of cells in the biofilm. Consistently, the results of RT-qPCR revealed that the expression of genes associated with biofilm formation was downregulated by S-342-3 (*P* < 0.05). However, we found that treatment with S-342-3 resulted in a significant decrease in the expression of global regulatory genes *agrA* and *sarA* (*P* < 0.05), which play a key role in promoting cell surface attachment and PIA production in *S. aureus* biofilms. Also importantly, we experimentally proved that the S-342-3 was not toxic to A549 alveolar epithelial cells and the *Galleria mellonella* larvae. Collectively, these results suggest that the S-342-3 may be a promising anti-biofilm drug candidate with excellent biosafety, which can be further explored for its practical application in health care.

**IMPORTANCE:**

Biofilms are an important virulence factor in *Staphylococcus aureus* and are characterized by a structured microbial community consisting of bacterial cells and a secreted extracellular polymeric matrix. Inhibition of biofilm formation is an effective measure to control *S. aureus* infection. Here, we have synthesized a small molecule compound S-342-3, which exhibits potent inhibition of biofilm formation in both MRSA and MSSA. Further investigations revealed that S-342-3 exerts inhibitory effects on biofilm formation by reducing the production of polysaccharide intercellular adhesin and preventing bacterial adhesion. Our study has confirmed that the inhibitory effect of S-342-3 on biofilm is achieved by downregulating the expression of genes responsible for biofilm formation. In addition, S-342-3 is non-toxic to *Galleria mellonella* larvae and A549 cells. Consequently, this study demonstrates the efficacy of a biologically safe compound S-342-3 in inhibiting biofilm formation in *S. aureus,* thereby providing a promising antibiofilm agent for further research.

## INTRODUCTION

*Staphylococcus aureus* is a Gram-positive human pathogen causing numerous different sorts of diseases ranging from simple soft tissue infections to life-threatening diseases such as bloodstream infections, toxic shock syndrome, and necrotizing pneumonia, which represent a tremendous burden on the whole healthcare system (1). Due to the prevalence of multidrug-resistant strains, particularly methicillin-resistant *S. aureus* (MRSA), the treatment of their infections has become more and more difficult (2). More seriously, an increasing number of reports suggested that MRSA has evolved multiple mechanisms to evade attacks of last-line antimicrobial agents such as vancomycin (3).

Biofilm formation is one of the strategies for *S. aureus* to evade host immunity and resist antibiotic therapy (4). According to other studies, bacteria in biofilms are 10–1,000 times more resistant to antibiotics than corresponding planktonic bacteria (5, 6). Biofilm is an adherent microbial community, which is mediated by bacterial cells embedded into a protective extracellular polymeric matrix. The extracellular polymeric substances are composed of polysaccharide intercellular adhesin (PIA), eDNA, proteins, and other biomolecules (7, 8). Among them, the PIA is one of the most studied components and is encoded by the *ica* operon (9, 10). The initial attachment represents a crucial first step in the process of biofilm formation, which is positively regulated by genes such as *fnbB*, *eno*, *psm*, and *ebps*. Many regulators, such as *agr* and *sarA*, are also vital during the biofilm development process (11). The *agr* quorum-sensing system participates in regulating the density of cells and plays a crucial role in the processes of initial attachment and biofilm disassembly (12, 13). The *sarA* is a positive regulator of biofilm formation involved in regulating the production of PIA and adhesins (14, 15). Therefore, drugs that can inhibit the expression of biofilm genes may also be effective in inhibiting the pathogenicity of *S. aureus*.

Biofilm-associated infections are very difficult to treat with the existing antibiotics. Hence, we need to develop new effective drugs to address this problem. The S-342-3 is a small-molecule compound that is the product of the alkylations of indoles and maleimides containing different substituents. Indole and amide-related compounds have shown strong and effective anti-biofilm activity against MRSA in our previous studies (16). In particular, amide-containing compounds are widely present in various drugs (17). Studies have shown that indolylmaleimide compounds possess unique structural features that endow them with a wide range of biological activities (18). However, due to its ability to inhibit the signal transduction pathway of tissue cells, it is extremely toxic in clinical trials (19). Therefore, modifying the structure of indolylmaleimide compounds holds significant clinical implications. By adding a series of substituents, we synthesized the derivative compound S-342-3 from indolylmaleimide. Based on the biological activities of indolymaleimide compound, we speculate that S-342-3 may exhibit anti-virulence activity against *S. aureus*.

In the present study, we aimed to investigate the anti-biofilm efficacy of the small-molecule S-342-3 to evaluate the potential of S-342-3 for treating persistent *S. aureus* infection.

## MATERIALS AND METHODS

### Bacterial strains and growth conditions

*S. aureus* strains used in the study are listed in Table 1. All strains used in the experiments were clinical isolates and isolated from patients at the First Affiliated Hospital of Wenzhou Medical University. The strain named JP21 belongs to the methicillin-sensitive *S. aureus* (MSSA), whereas JP5023 and MR383 belong to MRSA. JP21 and MR383 are classified as ST7, while JP5023 belongs to ST59. The medium of Trypticase soy broth (TSB, BD Biosciences, Franklin Lakes, NJ, USA) was used to culture all strains at 37°C with shaking (220 rpm).

**TABLE 1:** Bacterial strains used in this study

Strain	MIC (μg/mL）	Source	MLST
JP21	>128	Tissue	ST7
JP5023	>128	Blood	ST59
MR383	>128	Sputum	ST7

### Synthesis of S-342-3

S-342-3 was synthesized at the School of Pharmacy, Wenzhou Medical University. A mixture of N-pivaloyl indoles 1 (0.2 mmol), maleimides 2 (0.6 mmol), [RhCp*Cl_2_]_2_ (5 mol%), Ag_2_O (2 equiv.), AgSbF_6_ (20 mol%), and TFE (2 mL) were added to a glass tube and stirred at 80°C for 1–24 h. It was mixed with water and ethyl acetate after the reaction was stopped. The reaction mixture was extracted three times with ethyl acetate. The combined organic layer was washed two times with a small amount of water, followed by drying over anhydrous magnesium sulfate and filtration. The filtrate was evaporated under vacuum conditions, and the residue was subjected to purification via flash column chromatography using silica gel to yield the desired product S-342–3 (20).

### Determination of minimum inhibitory concentration (MIC)

S-342-3 was diluted by dimethyl sulfoxide (DMSO) (Boyun, Shanghai, China) to a concentration of 16 mg/mL. The minimum inhibitory concentration (MIC) of S-342-3 against strains was determined by broth microdilution method according to the recommendation of the Clinical and Laboratory Standards Institute (21). The strains were cultured in TSB overnight and adjusted with 0.9% sterile saline to a final MacFarland unit 0.5. Then bacterial suspension was 1:100 diluted in cation-adjusted Mueller-Hinton broth (CA-MHB). One hundred microliters of suspension and 100 µL medium containing a series of concentrations of S-342-3 were added to a 96-well microfilter plate. The DMSO was used as a control to exclude the influence of solvent. The plate was then incubated at 37°C for 16–18 h in an incubator and the well with the lowest concentration of S-342-3 that showed no visible bacterial growth was considered as the MIC. All assays were repeated three times.

### Crystal violet semiquantitatve biofilm assay

An overnight culture of strain was diluted 1:100 in TSB supplemented with 0.5% glucose (TSBG) containing different concentrations of drug (0–16 μg/mL), and then 200 µL of the diluted culture was added into each well of a 96-well plate. After incubation for 24 h at 37°C, the wells were washed three times with 200 µL of phosphate-buffered saline [PBS, Sangon Biotech (Shanghai) Co., Ltd.]. The biofilm was fixed with 100 µL of 99% methanol for 15 min and 200 µL of 1% crystal violet was used to stain the biofilm for 10 min. The wells were gently rinsed with running water, air dried, and observed for the density at OD_600_ nm after adding 30% acetic acid. The assay was performed in triplicate.

### Growth assay

To determine the effect of S-342-3 on MRSA and MSSA, growth assays were performed. Briefly, the strains were streaked on blood agar plates and inoculated into TSB to grown overnight at 37°C in a shaker. The bacteria suspension with a concentration of 0.5 MFU was prepared with 0.9% sterile saline. The suspension was performed at 1:200 dilution into TSB medium containing S-342-3 at a concentration of 4 µg/mL. A total of 200 µL mixtures were added to a sterile bioscreen honeycomb plate. To exclude the influence of DMSO on bacterial growth, an equivalent concentration of DMSO to the S-342-3 was added as a control and TSB as a negative control. In the experiment, an automated microbial growth curve analyzer (OY Growth Curve, Finland) was used to measure the optical density at 600 nm every 1 h until 24 h. The bacterial solution after 30 h was taken and the number of viable bacteria was counted after dilution of the plate. After that, spot 5 µL different dilutions of the bacterial solution onto the blood plate and incubated for 16 h. The test was performed three times.

### Confocal laser scanning microscopy

The mid-log phase bacteria suspension was 1:200 diluted into TSBG with the S-342-3 of 4 µg/mL and 1 mL was seeded into 15 mm glass-bottomed cell culture dishes (NEST, Wuxi, China). After the dishes were incubated for 24 h at 37°C, the dishes were washed three times with PBS, after which the supernatant was discarded and the dishes were air dried at room temperature. Add 500 µL mixtures of SYTO-9 (0.02%, Thermo Fisher Scientific, Waltham, MA, USA) and PI (0.067%, Thermo Fisher Scientific) to stain the biofilm in the dark for 30 min. The stained biofilms were visualized by confocal laser scanning microscopy (CLSM) (TCS SP5; Leica, Wetzlar, Germany).

### Effect of S-342-3 on bacterial adhesion

The experiment was performed according to the previous study (16) . In brief, 20 µL of overnight bacterial cultures was added to the wells of a six-well plate containing 2 mL TSBG medium supplemented with S-342-3 at a concentration of 4 µg/mL, the drug-free wells served as controls. The plates were incubated at 37°C for 3 h for the bacteria to attach. The wells were washed with sterile PBS and removed the planktonic cells, 1 mL PBS was added, then the biofilm was scraped off with a cell scraper and transferred to a 1.5-mL tube. Count of colonies on the second day after coating on the blood plate with different dilution multiples.

### Cell cytotoxicity

The cytotoxicity of S-342-3 in A549 cells was evaluated in a CCK-8 assay according to the guidelines of the manufacturer’s instructions. A549 cells were incubated in a 96-well plate at a density of 1 × 10^4^ cells per well with Dulbecco’s modified Eagle’s medium [DMEM, Thermo Fisher Biochemical Products (Beijing) Co., Ltd.] and incubated for 12 h at 37°C. Next, the supernatant in each well was discarded and treated with DMEM containing a final concentration of 4 µg/mL of S-342-3. The well without drugs was used as a control. After incubation for 24 h, each well was washed two times with sterile PBS. The cells were then incubated with 100 µL DMEM and 10 µL CCK-8 reagent for 1–2 h at 37°C in the dark. Finally, the viability of cells was quantified by measuring the absorbance at 450 nm; the results were performed as the percentage of viable cells compared to the control cells.

### *In vivo* efficacy of S-342-3

In previous studies, *G. mollonella* larvae are highly suitable as an alternative nonmammalian animal model for evaluating the toxicity and efficacy of antimicrobial agents *in vivo* (22). The toxicity of the compound *in vivo* was determined by the greater wax moth larvae assay. Briefly, larvae weighing between 0.2 and 0.25 g were taken for our experiments and 10 randomly chosen healthy larvae were used per group in each experiment. The compound was diluted with sterile normal saline to a concentration of 4 µg/mL. An equivalent concentration of DMSO to the 4 µg/mL S-342-3 was used as a control to exclude the effect of solvent on larvae. The larvae were injected with 10 µL of the mixture into the last left proleg and incubated in plastic petri dishes at 37°C in the dark. Survival was monitored every 12 h for 3 days and the percent of survival for each group was calculated. In this experiment, the group was injected with sterile saline as a control. The experiment was repeated three times.

### Enzyme-linked dot immunoblot assay for polysaccharide intercellular adhesin

The overnight cultures were 1:100 diluted in 3 mL TSBG containing a concentration of 4 µg/mL S-342-3 and then added into a six-well plate at 37°C. The wells were without drugs as controls, and each well was washed with sterile PBS. The 500 µL of 0.5 M EDTA [PH 8.0, Sangon Biotech (Shanghai) Co., Ltd.] was added into wells and plates were placed on the ice for 1 h. After that, the biofilm was scraped and transferred to a 1.5-mL microcentrifuge tube. The tubes were incubated at 100°C for 10 min and centrifuged at 12,000 rpm for 5 min. The 40 µL supernatant was removed to another centrifuge tube and 20 µL proteinase K (20 mg/mL) was added and inoculated at 37°C for 2 h. Then, 10 µL extract sample was spotted onto the nitrocellulose filter (NC) membrane which was soaked with water. When the extract was completely absorbed, 3.5% bovine serum albumin (BSA) (Biosharp, Beijing, China) was used to block the membrane at 4°C overnight. Subsequently, the membrane was incubated in Wheat Germ Agglutinin-HRP (WGA-HRP) solution for 1 h at 37°C, and the membrane was washed three times with PBST [Sangon Biotech (Shanghai) Co., Ltd.] fully and visualized with enhanced chemiluminescence (ECL) (Affinity Bio, San Francisco, CA, USA).

### Quantitative RT-PCR analysis

*S. aureus* strains were inoculated with TSB with or without 4 µg/mL S-342-3 to shake for 16 h at 37°C. Total RNA was isolated according to the manufacturer’s protocol [Spin Column Bacteria Total RNA Purification Kit and Sangon Biotech (Shanghai) Co., Ltd.]. After RNA samples were reversed transcribed into cDNA, a quantitative real-time PCR (qPCR) was performed by using a SYBR Green master mix (Takara, Tokyo, Japan). The primer pairs used in RT-qPCR experiment are listed in Table 2. The *gyrB* gene was used as an internal control, and the candidate gene expressions were calculated using 2^−△△Ct^ values as previous study (16). This experiment was performed in biological triplicates with three technical replicates.

**TABLE 2:** Primes used in this study

Primer name	Sequence (5′−3′)
*gyrb*-RT-F	GGTGGCGACTTTGATCTAGC
*gyrb*-RT-R	TTATACAACGGTGGCTGTGC
*icaA*-RT-F	GTTGGTATCCGACAGTATA
*icaA*-RT-R	CACCTTTCTTACGTTTTAATG
*icaB*-RT-F	CCTATCCTTATGGCTTGATGA
*icaB*-RT-R	CATTGGAGTTCGGAGTGA
*icaC*-RT-F	TACTGACAACCTTGAATTACCA
*icaC*-RT-R	AATAGCCATACCATTGACCTAA
*icaD*-RT-F	CCAGACAGAGGGAATACC
*icaD*-RT-R	AAGACACAAGATATAGCGATAAG
*fnbB*-RT-F	GCGAAGTTTCTACTTTTG
*fnbB*-RT-R	CAACCATCACAATCAACA
*eno*-RT-F	CTCCAATTGCATTCCAAG
*eno*-RT-R	GCATCTTCAGTACCTTCA
*fib*-RT-F	GTGCTTTACGGTGTGTTG
*fib*-RT-R	CTGCTATTAGTTTAACGGTATCAA
*ebpS*-RT-F	GTGTGATGATTCGACTTG
*ebpS*-RT-R	CAGGATACAATAGAGAATACG
*agrA*-RT-F	GCAGTAATTCAGTGTATGTTCA
*agrA*-RT-R	TATGGCGATTGACGACAA
*sarA*-RT-F	AAACCCTGAATTTGAATG
*sarA*-RT-R	GATATTACATCTGCTCCT
*psmα*-RT-F	ATGGAATTCGTAGCAAAATTATTC
*psmα*-RT-R	TAGTTGTTACCTAAAAATTTACC
*psmβ*-RT-F	CCTAGTAAACCCACACCG
*psmβ*-RT-R	GCTGCACAACAACATGATA

### Statistic analysis

All the experimental data were analyzed by GraphPad Prism 7 and presented as mean ± SD (standard error of the mean). The one-way ANOVA or Student’s *t* test was used for comparison among multiple groups. The difference was considered significant with a *P* value of <0.05.

## RESULTS

### Antibacterial activity of S-342-3 and inhibitory effect on biofilm formation of *S. aureus*

**Fig 1:**
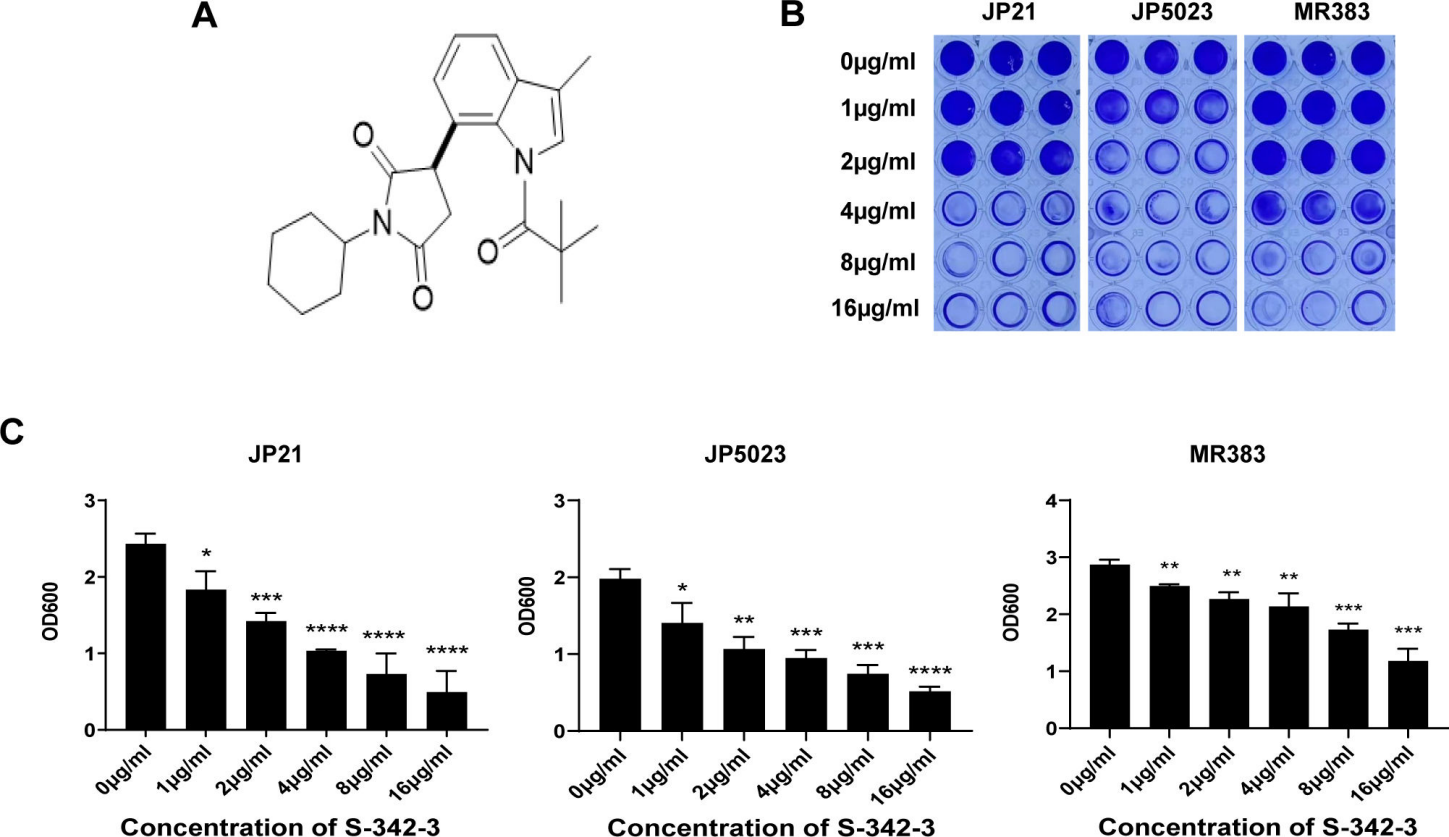
Anti-biofilm activity of S-342-3. (**A**) The chemical structure of compound S-342-3. (**B**) Biofilm formation in the 96-well plate. (**C**) The optical density (OD) value measured at 600 nm and there were significant differences with the control group for three strains. *P < 0.05, **P < 0.01, ***P < 0.001, ****P < 0.0001.

We assessed the antibacterial activity of S-342-3 against JP21, JP5023, and MR383. The MIC of S-342-3 against all tested isolates was found to be >128 µg/mL, indicating the compound was devoid of antibacterial activity. The intricate structure of biofilms provides a protective shield for bacteria, making them resistant to antimicrobial agents and difficult to remove (18). The crystal violet semi-quantitative method was used to explore the effect of S-342-3 on *S. aureus* biofilm formation. When treated with a sub-MIC of S-342-3 at 4 µg/mL, the biofilm of the JP21, JP5023, and MR383 were reduced compared with the untreated group (57.43%, 52.14%, and 25.49%, respectively) (Fig. 1B and C). Following exposure to a range of sub-MICs, S-342-3 inhibited the biofilm formation of all isolates in a dose-dependent manner.

### Influence of S-342-3 on the growth of methicillin-resistant and methicillin-sensitive *Staphylococcus aureus* strains

To ensure that the inhibitory action of S-342-3 was specifically directed towards biofilm formation, and not merely a consequence of restricting bacterial growth, we chose the sub-inhibitory concentration of 4 µg/mL to evaluate the impact of the compound on bacterial growth. At the tested concentration, according to the growth curve for 24 h, the amount of bacteria at the late logarithmic growth phase was consistent with the drug-free group (Fig. S1A). At the same time, we demonstrated this by counting viable bacteria (Fig. S1B and C). It indicates that, at this concentration level, the drug is capable of preventing biofilm formation without affecting bacterial growth.

**Fig 2:**
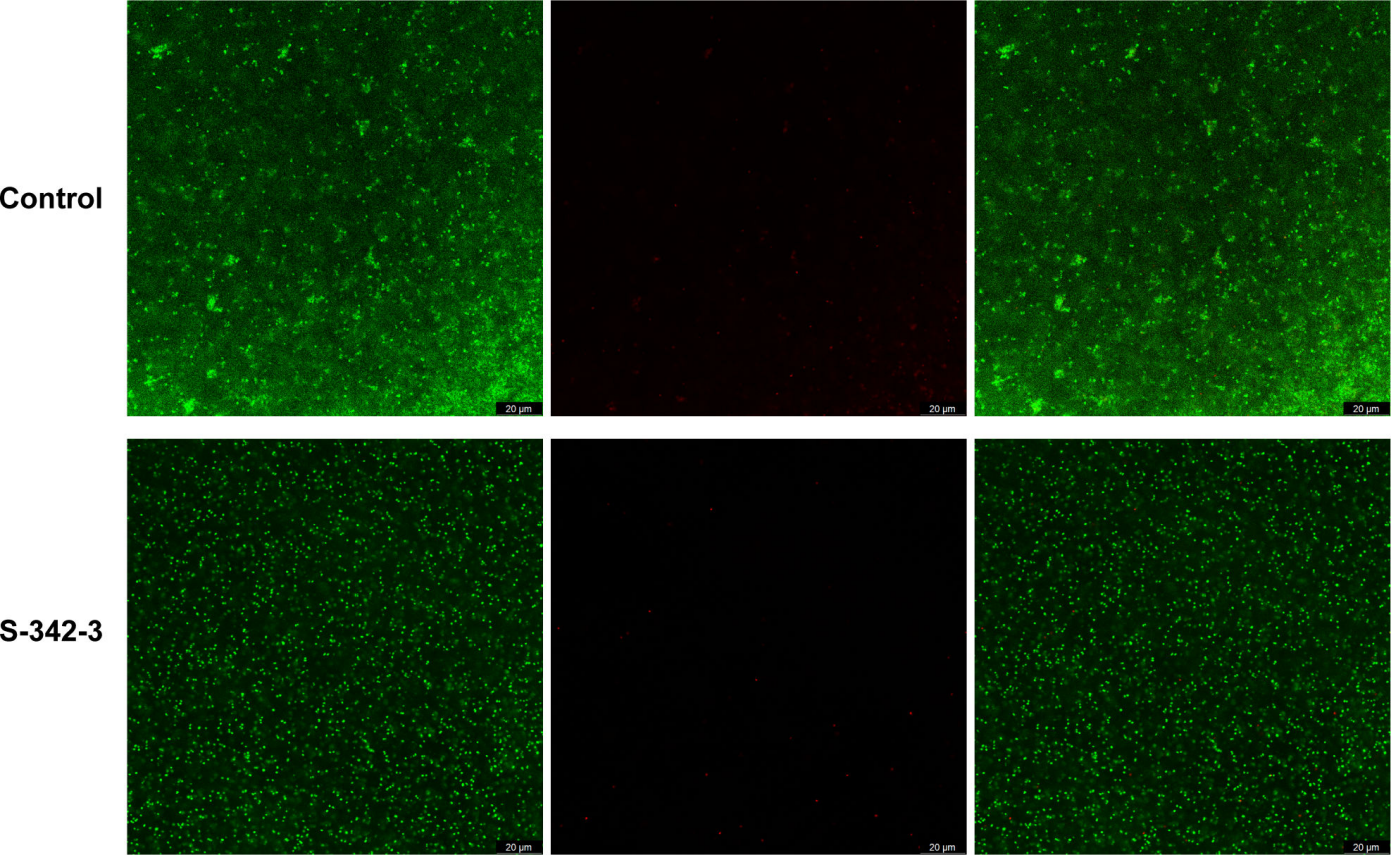
Structural characteristic of biofilm was observed by CLSM after treated with 4 µg/mL S-342-3. The live (left) /dead (middle) *Staphylococcus aureus* strain viewed under confocal microscopy, and merged images (right).

In our subsequent investigation, we utilized confocal CLSM to examine the biofilm architecture of MR383. After exposure to the compound at 4 µg/mL, the architecture of the biofilm was found to be looser and more contained fewer live and dead cells (Fig. 2). These results also demonstrated the antibiofilm activity of S-342-3.

### S-342-3 affects the initial adhesion of *Staphylococcus aureus*

**Fig 3:**
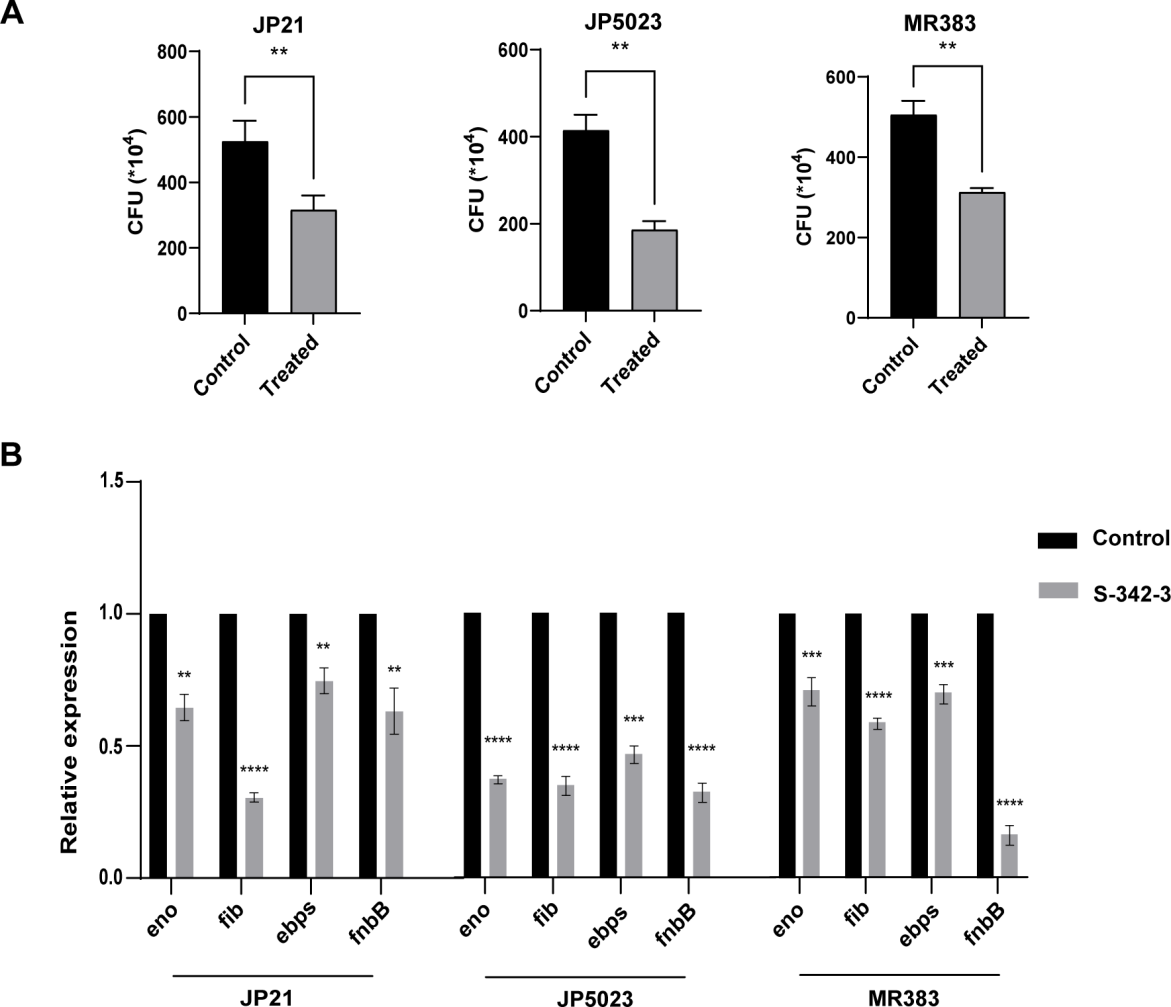
Effect of 4 µg/mL S-342-3 on the adhesion ability of *S. aureus*. (**A**) The adhesion of bacteria was determined by CFU count assay. (**B**) Alterations in genes associated with attachment after treated with S-342-3. **P < 0.01, ***P < 0.001, ****P < 0.0001.

Biofilm formation is a multistep process that begins with attachment to the surface (23). Through initial adhesion assay, we examined the impact of a sub-inhibitory concentration of S-342-3 (4 µg/mL) on the initial adhesion stage of MRSA and MSSA biofilm formation. The results indicated that S-342-3 significantly suppressed the adhesion of JP5023, JP21, and MR383 (Fig. 3A). Moreover, the expression of genes related to cell adhesion, including *eno*, *fib*, *ebps*, and *fnbB*, were also significantly decreased when exposed to 4 µg/mL of S-342-3 (Fig. 3B).

### S-342-3 inhibits the production of PIA in *S. aureus*

**Fig 4:**
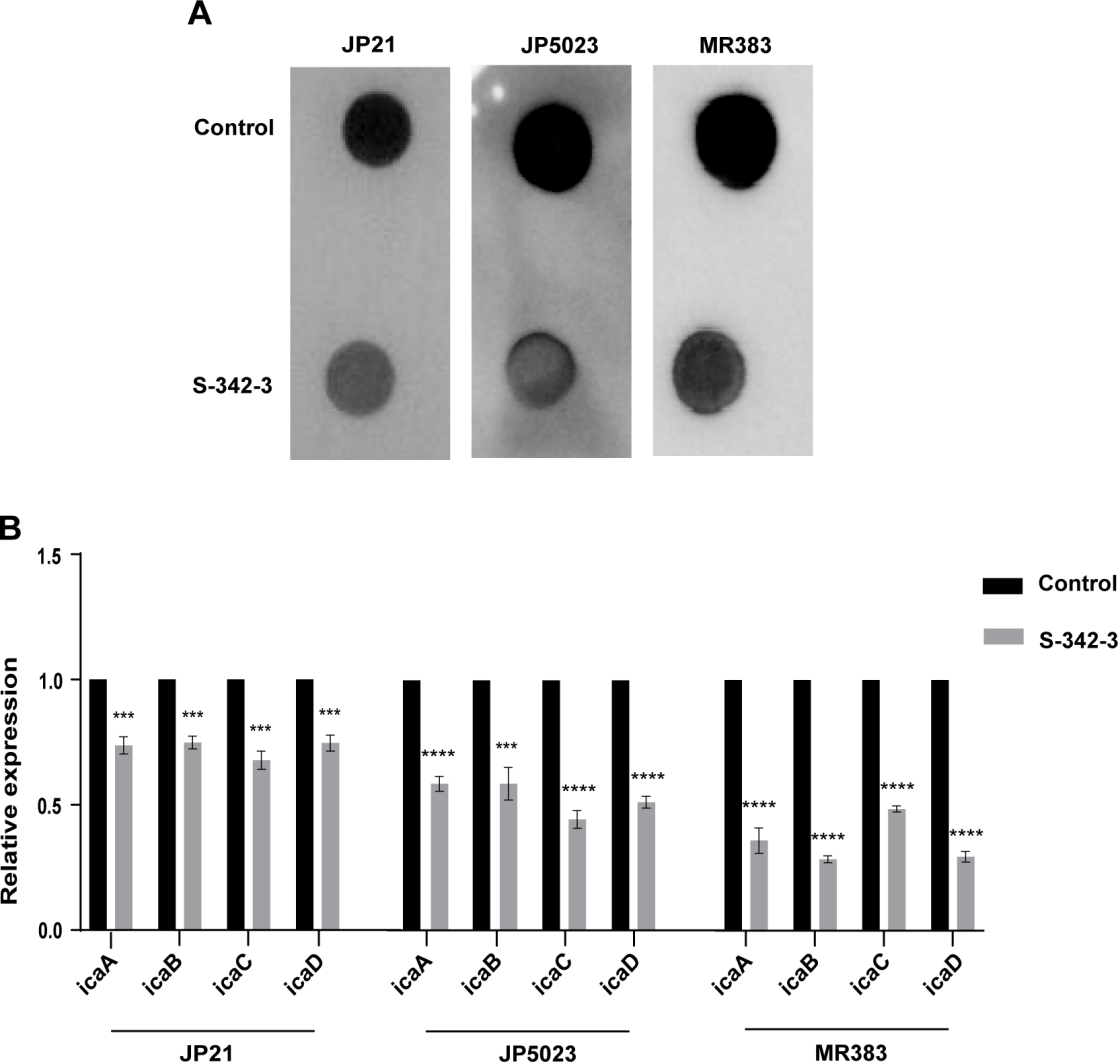
Effect of 4 µg/mL S-342-3 on the PIA production of *S. aureus*. (**A**) ELISA assay was used to semiquantify the biosynthesis of PIA. (**B**) The effect of S-342–3 on PIA-related genes expression.***P < 0.001, ****P < 0.0001.

As previously mentioned, PIA plays a significant role in mediating intracellular adhesion and providing structural integrity in biofilm formation (24). Subsequently, we investigate whether PIA production was altered in *S. aureus* in the presence of S-342-3. Compared to the untreated group, the intensity of spots on the membrane was reduced in both MRSA and MSSA after treatment with the compound (Fig. 4A). Consistently, the results of RT-qPCR showed that the expression levels of *icaADBC* were also downregulated (Fig. 4B). These findings indicate that the inhibition of *S. aureus* biofilm formation by S-342-3 may be attributed to a reduction in the production of PIA.

### Effects of sub-inhibitory concentration of S-342-3 on the expression of regulatory genes *agrA* and *sarA*

**Fig 5:**
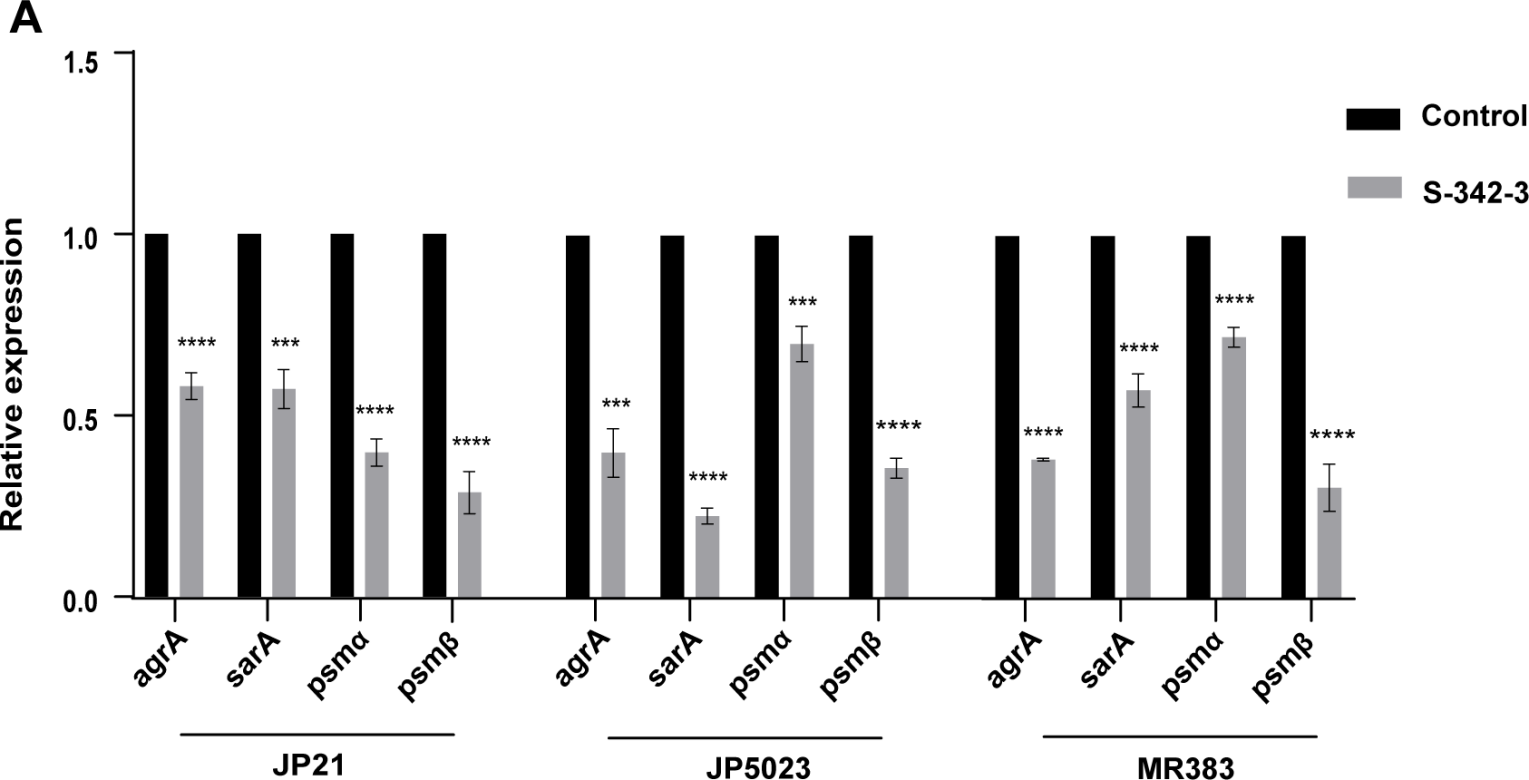
(**A**) RT-qPCR analysis of biofilm-related gene expression levels in *S. aureus* in the presence of 4 µg/mL S-342-3. ***P < 0.001, ****P < 0.0001.

As mentioned above, the sub-inhibitory concentrations of S-342-3 can effectively inhibit the formation of *S. aureus* biofilm. In order to elucidate the mechanism by which S-342-3 inhibits biofilm formation, we further investigated the expression of key regulatory genes involved in biofilm formation. The Agr quorum-sensing system plays a central regulatory role in *S. aureus* biofilm dynamics, with the *agrA* gene serving as an indispensable component of this system (25). As shown in Fig. 5A, when the strains were treated with 4 µg/mL of S-342-3 for 16 h, we found the expression of *agrA* significantly decreased. The expressions of *psm*α and *psmβ* were also decreased, which were regulated by regulatory gene *agr*. The sarA is a well-known global transcriptional regulatory factor by regulating the expression of *ica* gene to influence the formation of biofilm (26). In our study, all test strains exhibited varying degrees of decrease in the expression of the *sarA* gene. These results proved that sub-inhibitory concentrations of S-342-3 most likely inhibit the biofilm formation of *S. aureus* by preventing the expression of regulatory genes *agrA* and *sarA*.

### Evaluation of the toxicity of S-342-3 *in vitro* and *in vivo*

**Fig 6:**
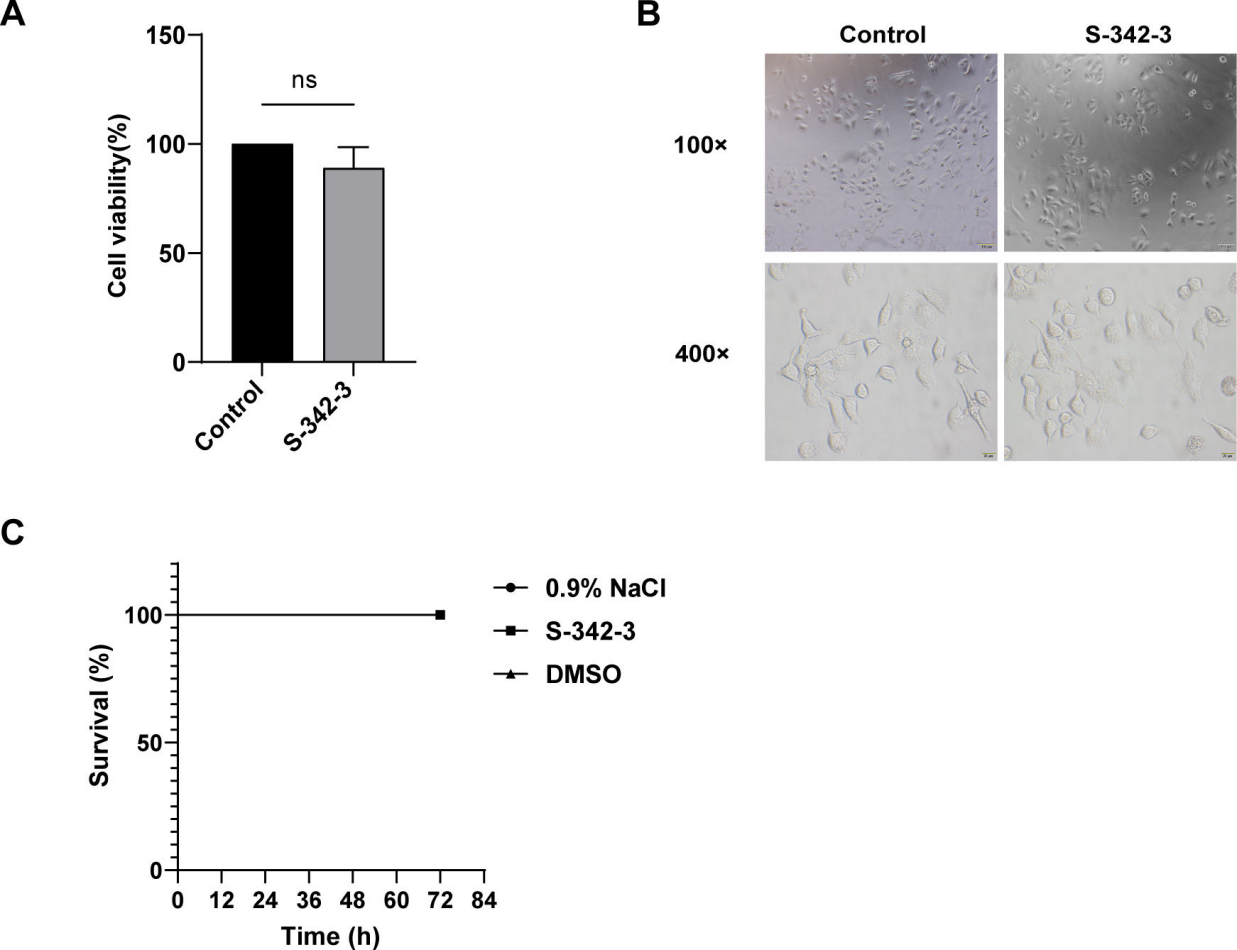
The toxicity of 4 µg/mLS-342-3. (**A**) S-342-3 cytotoxicity determined by CCK-8 assay. (**B**) Microscopic cell morphology. (**C**) *In vivo* toxicity of S-342-3 to the *G. mollonella* larvae.

Alveolar epithelial A549 cells with CCK-8 assay were used to evaluate the cytotoxicity of S-342-3. The results showed that there was no significant difference in absorbance at the wavelength of 460 nm between the compound S-342-3-treated groups and the vehicle-treated groups (Fig. 6A). This result demonstrates that the drug did not cause notable damage to cell viability. Next, the change of the morphological features of cell growth was observed under an inverted microscope and there was no obvious difference between the two groups (Fig. 6B). Additionally, the *G. mellonella* larvae infection model was conducted to gain a more comprehensive insight into possible toxicity *in vivo* caused by S-342-3. The administration of S-342-3 at a sub-inhibitory concentration of 4 µg/mL did not cause the death of the larvae (Fig. 6C). Thus, these results indicated that S-342-3 was non-toxic in both *in vitro* and *in vivo* settings.

## DISCUSSION

Biofilm poses numerous challenges in the medical field as well as various other industries. Bacteria have the ability to form biofilms on the surface of implants, rendering them more difficult to detect and treat than planktonic counterparts (27). The demand for novel antibiofilm agents and the research to explore their molecular mechanism have significantly increased in recent years. For instance, the LCG-N25 is a small molecule and it has been shown to have a potent activity against oral streptococcal biofilm (28). In this study, we identify a novel small-molecule compound S-342-3 that exerted a strong inhibitory activity against biofilm formation in both MRSA and MSSA clinical isolates.

Biofilm formation is a complex and multifactorial process. The process typically involves several major steps, including initial attachment, proliferation, maturation, and dispersion (29, 30). Notably, preventing bacterial adhesion to the surface of an implant is one of the most critical strategies for avoiding biofilm formation (31). To explore whether S-342-3 exposure affects the ability to initiate and develop the biofilm of *S. aureus*. We assess the effect of S-342-3 on bacterial adhesion. The results of our study showed that a concentration of 4 µg/mL of S-342-3 effectively inhibited bacterial initial adhesion and repressed the expression of adhesion-related genes, including *fib*, *fnbB*, *ebps,* and *eno*.

According to the biofilm matrix, the mechanisms of biofilm formation can be classified as either PIA-independent or PIA-dependent (32). Previous studies found that the biofilm formation of MSSA was PIA-dependent, and the MRSA was PIA-independent (33, 34). In contrast, all strains whether MSSA or MRSA synthesize large amounts of PIA in our study. According to previous research, the synthesis of PIA is dependent on the *ica* operon, which contains four genes (*icaADBC*) and a divergently transcribed repressor (*icaR*) (15). In our study, we found that S-342-3 could significantly inhibit the production of PIA. This conclusion was also proved by the RT-qPCR which showed S-342-3 repressed the expression of genes *icaA*, *icaD*, *icaB*, and *icaC* at the mRNA level.

The final stage in the development of *S. aureus* biofilm is characterized by the dissemination of individual bacterial cells from the biofilm to distinct locations, resulting in the establishment of new biofilms (35, 36). Phenol soluble modulins (PSMs) are a group of molecules that participate in shaping staphylococcal biofilm structure and regulate the maturation processes of biofilm, specifically the detachment of biofilm and dissemination from biofilms *in vivo*. It is grouped in α-type (PSMα and PSMγ) and β-type (PSMβ) peptides (37, 38). Research has shown that pyrancoumarin derivative LP4C effectively attenuates MRSA biofilm development by downregulating the expression of the genes *psmα* and *psmβ* (39). In this study, we found that the genes *psmα* and *psmβ* were also notably reduced after the treatment of S-342-3. The Agr system is the most studied of the QS system, includes the two transcriptional units, RNAII and RNAIII (2). The *agrA* is an essential transcription factor for RNAIII. Although RNAIII is the primary effector of the QS system, some genes are directly regulated by *agrA*. For example, the transcription of *psmα* and *psmβ* genes in *S. aureus* is directly driven by *agrA* (15, 40). The Agr system positively regulates the expression of *psm* by directly binding AgrA to the promoter region of the *psm* operon (41, 42). Consistent with the above, our results indicated that the expression of *psms* gene was downregulated while the expression of *agrA* decreased. Aside from QS system, the SarA regulatory system is also involved in the regulation of biofilm formation (43). The role of SarA regarding biofilm formation is to repress the production of extracellular proteases and PIA, and induce the expression of *fnbA*, *fnbB*, and *clfA* (determinants of *S. aureus* surface adhesins) (44). In our study, we found that exposure to sub-inhibitory concentrations of S-342-3 resulted in a reduction in the expression of the regulatory gene *sarA*. In a word, our experimental results showed that S-342-3 inhibited *S. aureus* biofilm formation by negatively regulating the expression of the regulatory genes *agrA* and *sarA*, so that to prevent the initial adhesion of bacteria and the production of PIA. Importantly, our data also demonstrated that S-342-3 was not toxic to A549 cells and *G. mellonella*.

In conclusion, our study has demonstrated that compound S-342-3 possesses significant antibiofilm activity and displays a favorable drug safety profile. These results suggest that compound S-342-3 holds great potential as a promising candidate for addressing biofilm-related issues in future clinical applications and may represent a novel therapeutic option for the treatment of *S. aureus* infections. A limitation of our research is that the effects of S-343-2 on the biofilm of bacteria other than *S. aureus* were not further studied. Another limitation is the bacteria used in our study were all clinical strains and the number was small, which makes the results not generalizable.

## SUPPLEMENTAL MATERIAL

Supplemental figure legend spectrum.01596-23-s0001.docx Legend for Fig. S1.

Fig. S1 spectrum.01596-23-s0002.tif Effect of 4 μg/ml S-342-3 on the growth of S. aureus.

ASM does not own the copyrights to Supplemental Material that may be linked to, or accessed through, an article. The authors have granted ASM a non-exclusive, world-wide license to publish the Supplemental Material files. Please contact the corresponding author directly for reuse.
