# Supplementary material for: A novel small-molecule compound S-342-3 effectively inhibits the biofilm formation of Staphylococcus aureus
Source: Microbiol Spectr. 2023 Oct 11;11(6):e01596-23. doi: 10.1128/spectrum.01596-23 (PMC10714762; doi:10.1128/spectrum.01596-23)
Supplement: Supplemental figure legend — Legend for Fig. S1. [file spectrum.01596-23-s0001.docx]

Supplementary Figure S1: Effect of 4 μg/ml S-342-3 on the growth of *S. aureus.* (A) Growth curves of three strains treated with S-342-3, and trypticase soy broth (TSB) as a blank control, the group with dimethyl sulfoxide (DMSO) were used to exclude the influence of solvent on bacterial growth. (B) Colony count of three strains on blood agar plates after thirty hours incubation and (C) spotting a serial dilution on blood agar plates.
