# Supplementary figures and images for: A novel small-molecule compound S-342-3 effectively inhibits the biofilm formation of Staphylococcus aureus
Source: Microbiol Spectr. 2023 Oct 11;11(6):e01596-23. doi: 10.1128/spectrum.01596-23 (PMC10714762; doi:10.1128/spectrum.01596-23)

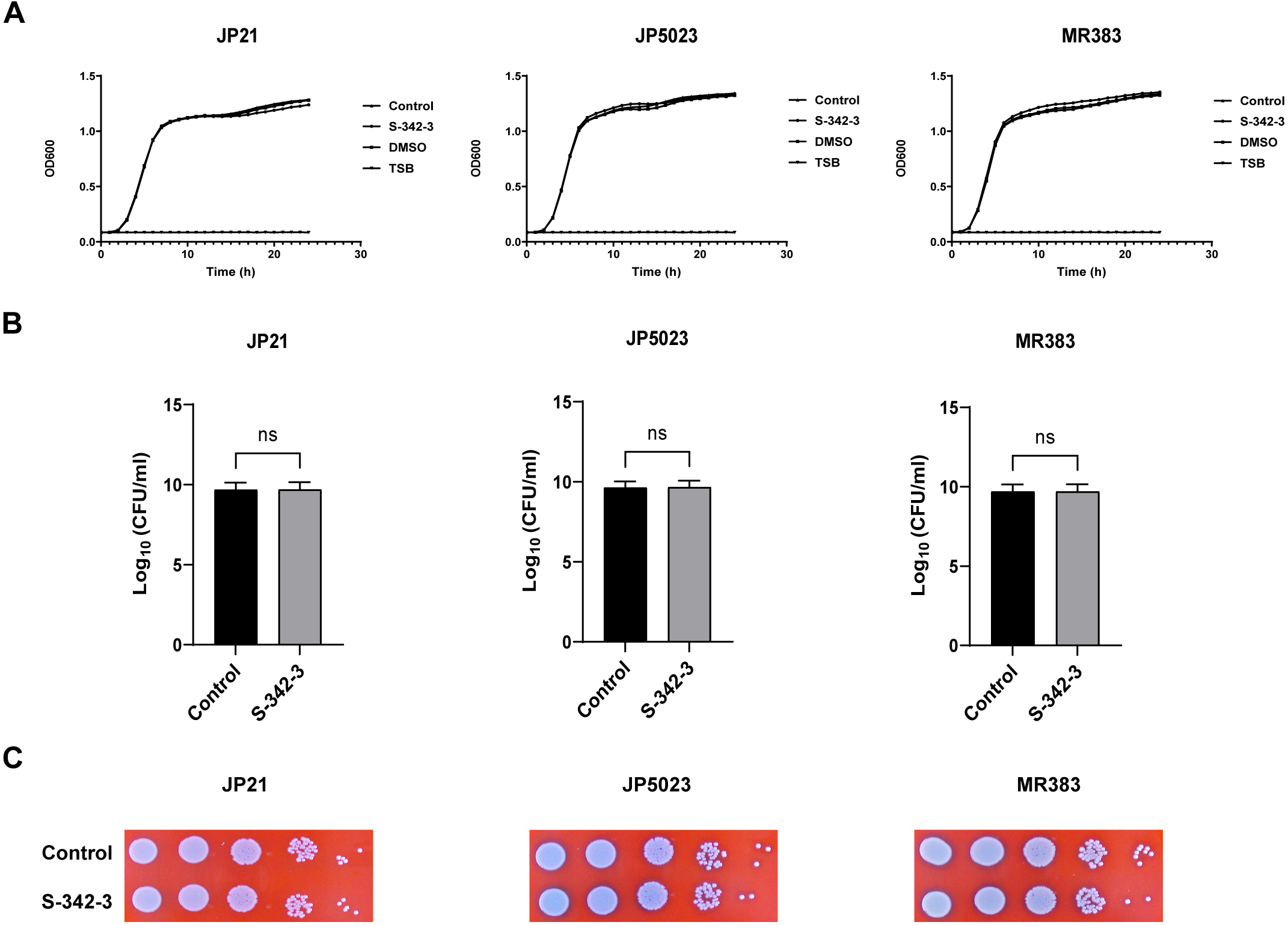

Supplement: Fig. S1 — Effect of 4 μg/ml S-342-3 on the growth of S. aureus. [file spectrum.01596-23-s0002.tif]
